# High-Pressure Injection Injury of the Face: A Case Report on Presentation and Management

**DOI:** 10.5811/cpcem.2020.3.45637

**Published:** 2020-04-23

**Authors:** Edan Zitelny, Blake Briggs, Rachel Little, David Masneri

**Affiliations:** * Wake Forest School of Medicine, Department of Emergency Medicine, Winston-Salem, North Carolina; † Wake Forest University Baptist Medical Center, Department of Emergency Medicine, Winston-Salem, North Carolina

**Keywords:** compartment, pressure, injection injury, plastics, face

## Abstract

**Introduction:**

High-pressure injection injuries have been chronicled for decades.^1^ These injuries often affect distal extremities as they are most commonly involved in workplace accidents.^1^ However, we discuss a young male with a paint-gun injection injury to his face.

**Case Report:**

We discuss the case of a young man presenting to the emergency department after high-pressure injection injury to the face. He eventually underwent extensive debridement of the face. We discuss differences in caring for an injection wound to an extremity versus the face, including time sensitivity of treatment, initial stabilizing measures, and critical steps.

**Discussion:**

This case demonstrates a rare presentation of a high-pressure paint injection injury. This injury presented a unique surgical challenge where, despite compartment syndrome being less common, cosmetic outcome and infectious complication prevention remained critical priorities.

**Conclusion:**

While similarities exist in management of an injection injury to a limb, due to the rarity and deceptive appearance of this particular injury to the face, high suspicion along with urgent imaging and surgical consultation is warranted.

## INTRODUCTION

High-pressure injection injuries present unique pathology. They cause devastating deep tissue damage despite small, innocuous, and usually painless skin lesions most commonly occurring on the hands, fingers, or feet. The skin lesions associated with these injuries often dwarf the severity of the damage occurring below the surface, as foreign chemical irritants are introduced into a closed compartment.^2^ They are true surgical emergencies, requiring prompt surgical debridement, decompression, and thorough cleansing in the operating room to counter the risk of infection and compartment syndrome.^2^ While these injuries are often devastating, they are rare. According to a 1991 study, only one in 600 hand traumas include an injection injury under high pressure, with injection injuries to the feet even more rare.^3^ Without adequate treatment, surgical intervention, and proper follow-up care, morbidity is significant with amputation rates that reach as high as 48%.^3^

We report the case of a high-pressure injection injury to a unique anatomical location: the face. This case serves as a platform to explore the importance of the anatomical location of these injuries and how the location may guide treatment. Regardless of the misleading appearance of the wound and the low likelihood of compartment syndrome, high-pressure injection injuries to the face require a high level of suspicion and a low threshold for imaging and surgical consultation.

## CASE REPORT

**Image 1:**
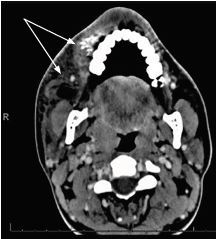
Computed tomography horizontal view demonstrating soft tissue hematoma/contusion and hyperdense material (arrowheads).

A 19-year-old male with no significant past medical history presented as a transfer to our Level I trauma center with a chief complaint of right facial injury. Eight hours prior to arrival to our emergency department (ED), the patient accidentally discharged a pressurized, spray-paint gun while attempting to troubleshoot his equipment at work. Due to a language barrier, an interpreter was used throughout the patient encounter. The history was obtained from the patient interview and a review of medical records provided by an outside hospital. The patient endorsed right facial pain and dysphagia. He denied any eye pain, dysarthria, decreased vision, or trouble breathing. At the outside hospital, the patient was administered a dose of ampicillin/sulbactam for broad-spectrum antibiotic coverage and underwent computed tomography of the face and sinuses with contrast (Image 1). The imaging demonstrated “soft tissue hematoma/contusion involving right premolar and premandibular soft tissue regions,” as well as “hyperdense material likely paint [and] no infraorbital injury.”

Review of systems demonstrated facial swelling, sinus pain, sinus pressure, and trouble swallowing. The rest of review of systems was negative.

**Image 2:**
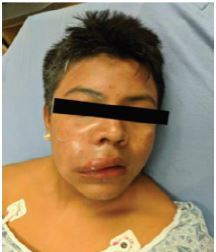
Demonstrated swelling and facial distortion on the right side from high-pressure paint injection injury.

On arrival to our ED, the patient was hemodynamically stable with normal vital signs and was in no acute distress. His physical exam revealed swelling of the right face extending from his cheek just below the eye to the right upper lip (Image 2). The affected area was tender to palpation. The ocular exam was normal. Laboratory studies revealed a mild leukocytosis but were otherwise normal. Otorhinolaryngology was consulted for bedside evaluation. Upon review of the patient, they attempted an incision and drainage at the bedside with normal saline flushes of the region and decided to admit him for observation and eventual operative management.

The patient underwent excision and debridement of the face. He received his nutrition via nasogastric tube. Antibiotics were given and all cultures were pan-negative for bacterial and fungal pathogens. Pain was managed with oxycodone and acetaminophen. He was discharged one week after admission without issue.

**CPC-EM Capsule**

What do we already know about this clinical entity?

High-pressure injection injuries, while appearing innocuous, are dangerous. Usually affecting distal limbs, they can cause compartment syndrome and necrosis.

What makes this presentation of disease reportable?

A pressure injection injury to the face is rare, with no current guidelines for management. This case may provide guidance in presentation and care.

What is the major learning point?

There are distinct differences in caring for an injection wound to extremity vs face, such as time sensitivity of treatment and initial stabilizing measures.

How might this improve emergency medicine practice?

Due to the rarity and deceptive appearance of this injury, high suspicion along with urgent imaging and surgical consultation is warranted.

## DISCUSSION

This case demonstrates a rare presentation of a high-pressure paint injection injury. The anatomical location of this injury presented a unique challenge to the management of this patient because the region of the injury was distinct from the more common presentations of paint injection injuries. Most often located on the hand or digits, paint injection injuries are surgical emergencies warranting debridement in a sterile setting and separation of fascial layers with the hope of preventing infections such as necrotizing fasciitis.^2^ The presentation of our patient differed significantly as his injection injury was located in the maxillofacial region lateral to the nose, medial to the temporomandibular joint, inferior to the orbits, and superior and anterior to the maxillary sinuses. This facial location of the injury greatly decreased the risk for compartment syndrome and allowed time for both the otorhinolaryngology and emergency medicine teams to properly evaluate the injury to determine the best course of action for this patient. This injury presented a unique surgical challenge where, despite compartment syndrome being less common, cosmetic outcome and infectious complication prevention remained critical priorities.

Initially the decision to attempt bedside incision and drainage (I&D) was made with the hope that irrigation would help flush out the region of the paint and alleviate the need for an invasive surgical debridement. It was quickly discovered that with such an elevated pressure of injection into the region, the paint was affixed very firmly to the surrounding tissue. Bedside irrigation was not thorough enough to address the extent of this injury. The patient was admitted to the hospital to the otorhinolaryngology service in stable condition. While official guidelines do not exist as to whether I&D is indicated for this injury, several case reports suggest that I&D is not successful in fully addressing the extent of paint injection injuries and surgery is indicated regardless of the injury location.^1^,^4^,^5^

The management of this patient was unique given the rarity, acuity, and severity of his presentation and the lack of concrete guidelines for a high-pressure paint injection injury to the face. Based on our literature review, there have only been two prior cases reporting high-pressure injection injury to the face, neither of which underwent acute surgical management from the ED. One case was observed and discharged as the high-pressure injury was only to the upper lip and the second case detailed granulomatous changes years later due to retained paint in the tissue causing a foreign body reaction.^6^,^7^

## CONCLUSION

High-pressure injection injuries to the face have a lower risk of compartment syndrome than high-pressure injection injuries to extremities; however, due to the rarity of reported cases, it is impossible to determine the risk of infectious complications, long-term neurovascular sequelae, or anatomical deformity. This case highlights the high level of suspicion emergency physicians must have despite the deceptive appearance of craniofacial high-pressure injuries and emphasizes the need for a low threshold for imaging and immediate surgical consultation.
